# Co-Expression Network Analysis of AMPK and Autophagy Gene Products during Adipocyte Differentiation

**DOI:** 10.3390/ijms19061808

**Published:** 2018-06-19

**Authors:** Mahmoud Ahmed, Jin Seok Hwang, Trang Huyen Lai, Sahib Zada, Huynh Quoc Nguyen, Trang Min Pham, Miyong Yun, Deok Ryong Kim

**Affiliations:** 1 Department of Biochemistry and Convergence Medical Sciences and Institute of Health Sciences, Gyeongsang National University School of Medicine, Jinju 527-27, Korea; ma7moud_sha3ban@hotmail.com (M.A.); cloud8104@naver.com (J.S.H.); tranghuyen20493@gmail.com (T.H.L.); s.zada.qau@gmail.com (S.Z.); nqh2412@gmail.com (H.Q.N.); phamminhtrang010895@gmail.com (T.M.P.); 2 Department of Bioindustry and Bioresource Engineering, College of Life Sciences, Sejong University, Seoul 05006, Korea; myyun91@gmail.com

**Keywords:** AMPK, autophagy, co-expression, microarrays, 3T3-L1, adipocyte, differentiation

## Abstract

Autophagy is involved in the development and differentiation of many cell types. It is essential for the pre-adipocytes to respond to the differentiation stimuli and may contribute to reorganizing the intracellulum to adapt the morphological and metabolic demands. Although AMPK, an energy sensor, has been associated with autophagy in several cellular processes, how it connects to autophagy during the adipocyte differentiation remains to be investigated. Here, we studied the interaction between AMPK and autophagy gene products at the mRNA level during adipocyte differentiation using public-access datasets. We used the weighted-gene co-expression analysis to detect and validate multiple interconnected modules of co-expressed genes in a dataset of MDI-induced 3T3-L1 pre-adipocytes. These modules were found to be highly correlated with the differentiation course of the adipocytes. Several novel interactions between AMPK and autophagy gene products were identified. Together, it is possible that AMPK-autophagy interaction is temporally and locally modulated in response to the differentiation stimuli.

## 1. Introduction

Autophagy is essential for the white adipocyte differentiation. The knockdown of *Atg5* and/or *Atg7* gene in the 3T3-L1 pre-adipocyte prevents its maturation upon the chemical induction [1,2]. Secondary to that, the pre-adipocyte fails to accumulate triglycerides and to form the fat droplets, which is a characteristic of mature white adipocytes [3]. This is yet to be reconciled with another observation in cells known to contain large quantities of lipids (e.g., hepatocytes) where autophagy takes part in lipid degradation [4]. AMP-activated protein kinase (AMPK), which can be activated at the low level of energy such as starvation, stimulates autophagy through the inhibition of the mTOR activity [5,6] and/or the direct phosphorylation of ULK1 [7,8]. Autophagy and AMPK regulate several aspects of the lipid metabolism and the cell response to changing energy levels. Therefore, the AMPK-autophagy interaction could be consequential in the context of adipocyte differentiation.

3T3-L1 pre-adipocyte is a mouse fibroblast with the potential to differentiate into a mature adipocyte when treated with the MDI differentiation induction medium (160 nM insulin, 250 nM dexamethasone, and 0.5 mM 1-methyl-3-isobutylxanthine) [9]. Upon induction, the pre-adipocyte undergoes multiple metabolic and morphological changes to reach maturation. Evidently, several of these changes can be observed at the transcription level of multiple adipogenic and lipogenic markers [10,11].

The aim of this work is to identify the potential AMPK-autophagy connections, in the broad sense of the pathways, that are both novel and consequential in the adipocyte differentiation, mainly using the weighted-gene co-expression network analysis (WGCNA) [12]. This approach focuses on identifying co-expressed pairs of genes across the differentiation stages, and enables the direct use of similar datasets to test and validate the findings even though they might be performed on different platforms.

In this study, we applied the WGCNA approach to a microarrays dataset of MDI-induced adipocyte at eight different time points corresponding to three differentiation stages. We identified two networks/modules among autophagy and AMPK gene products that were correlated with the differentiation course. By analyzing these modules in one of the datasets, we were able to specify several potential novel AMPK-autophagy interactions and connect these to candidate functions through the known annotations. Finally, we checked these findings in three independent datasets of similar design and found the networks/modules to be well preserved.

## 2. Results

### 2.1. Preparing Data and Annotations

First, we retrieved several microarray datasets from the Gene expression omnibus (GEO) [13]. We sought arrays of MDI-induced 3T3-L1 pre-adipocytes at three or more time points. After excluding the ones with varying designs and limited annotations, four datasets were included in the analysis (Table 1); one dataset (GSE34150) was chosen for the main analysis, and the rest were reserved for testing and validation. In GSE34150, the total RNA from 24 samples of MDI-induced pre-adipocytes were collected at eight different time points corresponding to three adipocyte differentiation stages (0 day, undifferentiated; two and four days, differentiating; 6–18 days, maturating). The initial quality assessment included checking the distribution of the intensities from all probes at the log scale, hierarchal clustering and multi-dimensional scaling analysis (MDS). Groups of samples from different stage of differentiation showed similar distributions, appropriate clustering and separation across the two different dimensions of MDS. Furthermore, to ensure the reliability of the analysis, we examined the expression of a number of differentiation and lipogenesis markers (Appendix A). The *Pparg*, *Cebpa* and *Lpl* genes, essential factors for adipocyte differentiation, were highly expressed in differentiating and maturing cells compared to the undifferentiated cells. Expression of most lipogenic genes (*Pparg, Cebpa, Lpl, Scd1, Scd2, Dgat1, Dgat2* and *Fasn*) was correlated with the development of 3T3-L1 pre-adipocytes into mature adipocytes.

The gene ontology (GO) terms: AMP-activated protein kinase activity (AMPK) and autophagy were used to identify 14 and 167 genes of known functions in the corresponding biological processes (BP), respectively. A total of 181 genes was used in the downstream analysis to limit the input to WGCNA, over-representation and defining novel interactions between and among AMPK and autophagy pathways. GO was also used to define the terms involving these interacting gene products and link them to known molecular functions (MF) and cellular components (CC). Appendix A contains a detailed discussion for the data inclusion criteria, quality assessment and obtaining the GO annotations.

### 2.2. Detecting Co-Expression Modules of AMPK and Autophagy Genes in Differentiating Adipocytes

**Figure 1:**
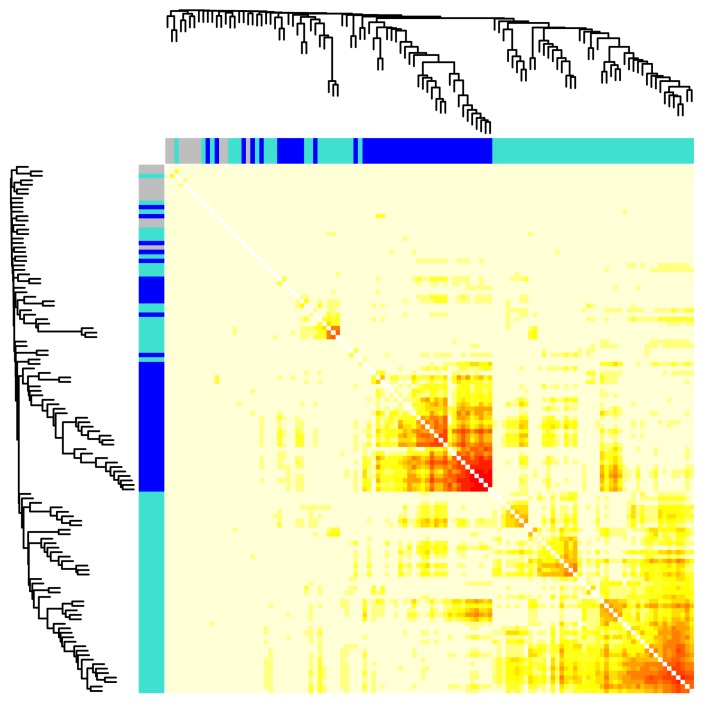
Clustering of AMPK and autophagy genes by their pairwise distances. Pairwise topological overlap matrix (TOM) similarities of AMPK and autophagy genes (*n* = 181) were calculated from their expression values in the GSE34150 dataset. Distances between each pair of genes were derived as 1 - TOM and shown as color values (small, red or large, yellow). A hierarchal tree and colored segments of the clusters were shown on the top and side.

Constructing co-expression networks is a multi-step process. First, the Pearson’s correlation coefficient was calculated between each pair of the genes of interest (*n* = 181) across all samples (*n* = 24). Second, these correlations were raised to the power 5 to obtain an adjacency matrix of all possible pairs. Third, the adjacency matrix was used to calculate the Topological Overlap Matrix (TOM) as a reliable similarity measure. Finally, TOM similarity between pairs of genes were used to calculate the weight of their connection in a network of all possible pairs and a distance (1 - TOM) to cluster the pairs into highly interconnected modules/colors (Figure 1). Appendix B provides a detailed discussion of the previous steps and the rationale for the different choices that were made in this analysis.

Among all possible pairwise correlations between the 181 genes of interest, two groups/modules of highly co-expressed gene products were formed (blue, 42; turquoise, 66), and the rest were unassigned (gray, 10). Genes that code for the subunits of the AMPK complex fell into different modules; four AMPK genes, *Prkaa2, Prkab2, Prkag2* and *Prkag3*, in the blue module along with 38 of the genes involved in autophagy; and two AMPK genes, *Prkab2 and Prkag1*, in the turquoise module together with 63 of the autophagy genes (Table 2). In the following sections, we describe the significance and the interactions of the individual members of these modules.

### 2.3. Correlating the Detected Modules to the Stage of Differentiation

**Figure 2:**
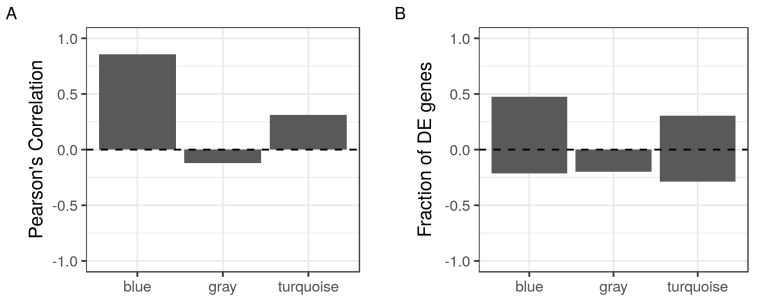
Correlations and over-representation of the detected modules in differentiation stages. The expression values of the members of the detected modules in the GSE34150 dataset (42, blue; 10, gray; and 66, turquoise) were used to calculate two representative summary statistics. (**A**) the first principal component (PC) across samples were correlated to the sample stages using Pearson’s correlation (bars); (**B**) the fraction of differentially expressed (DE) genes across differentiation stages (bars).

To establish the biological significance of these modules, we used the expression values of their individual members to calculate a representative summary—the first principal component (PC)—for each module. Then, we calculated the Pearson’s correlation coefficient for the first PC with the stage of differentiation (undifferentiated, differentiating or maturating) of all 24 samples. Both modules showed a reasonable correlation with sample stages (>0.8 for the blue and >0.3 for the turquoise module) (Figure 2A). In other words, the expression values of the members of the blue module, and to a less extent the turquoise, capture a lot of the observed differences between the cells as they progress from a differentiation stage to the next.

### 2.4. Testing the Over-Representation of the Modules over the Differentiation Course

Again, we considered the expression values of the individual members of each module to calculate the fraction of the differentially expressed genes (DE) across differentiation stages. Both blue and turquoise modules had a significant fraction of their member genes (>0.5) either up or downregulated at the differentiating or maturating stage compared to the control undifferentiated cell stage (Figure 2B). These fractions were significantly higher than the expected fractions of DE genes in randomly selected modules of the corresponding sizes. The calculated *p*-values were adjusted for multiple testing using the False Discovery Rate (FDR). Adjusted *p*-values less than 0.1 were considered significant.

### 2.5. Visualizing Modules and Identifying Novel AMPK-Autophagy Interactions

**Figure 3:**
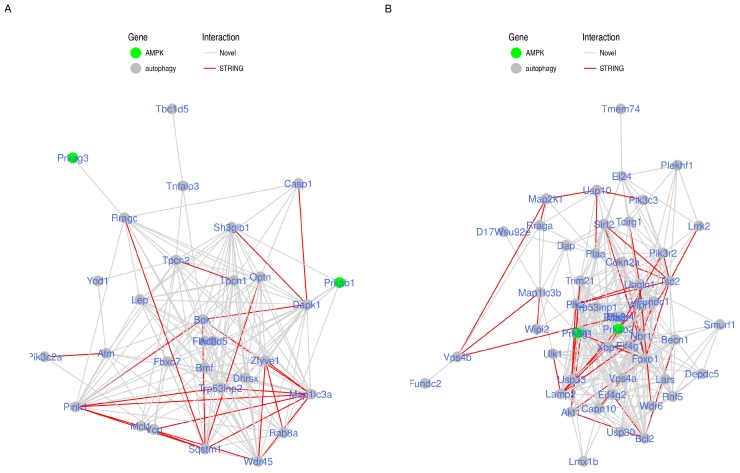
Network representation of the AMPK and autophagy modules. Members of the blue (**A**) and turquoise (**B**) modules are shown as a nodes. Each pair of nodes is connected by an edge if the corresponding pairwise topological overlap matrix (TOM) similarity/weight is above the threshold 0.1. Nodes are colored by gene category (AMPK, green or autophagy, gray). Edges are colored by type of interaction (STRING, red or Novel, gray).

To visually explore the detected modules, we treated each of their members as a node in a network graph. Nodes were divided into two networks based on the module to which they belong. Each pair of nodes was connected by an edge that has a weight calculated from the TOM similarity measure between the corresponding pair of genes. Edges with weights less than a minimum threshold (0.1) were excluded to obtain a less condense network (Figure 3). Evidently, some nodes did not share edges that passes this threshold and were not included in the network graph. In addition, nodes were labeled with the corresponding official gene symbol and colored as AMPK or autophagy genes; and the edges were colored by the novelty of the connection. The latter was determined mainly based on previous reports in the STRING database (textmining evidence), which is evidence extracted from abstracts of scientific literature.

By representing the modules in graphs, we were able to calculate different statistics to identify influential genes/nodes and important interactions/edges. Considering various centrality measures, we ranked the genes in each module by their influence on the modules (Table 3). *Trp53inp2, Map1lc3a, Wadr45, Pink1* and *Dapk1* genes were the most influential nodes in the graph of the blue module with a hub score more than 0.95, while *Foxo1, Dcn* and *Xbp1* genes had the highest scores in the turquoise module. Edges between AMPK and autophagy nodes that were not previously reported in the STRING database (text-mining evidence) were considered novel potential interactions (Table 4). The protein kinase AMP-activated non-catalytic subunit beta 1 (Prkab1) showed a potential interaction with several autophagy gene products including Bcl2-modifying factor (Bmf), death associated protein kinase 1 (Dapk1), Ras-associated protein Rab8a (Rab8a), SH3 domain, GRB2-like, endophilin B1 (Sh3glb1) and transformation related protein 53 inducible nuclear protein 2 (Trp53inp2) as part of the blue module. Similarly, the gamma subunit 1 (Prkag1) in the turquoise module revealed a novel binding ability to some well-known autophagy-related gene products such as Becn1, Fundc1, Lamp2 or Map1lc3b and also showed a novel interaction with some other gene products including calpain-like cysteine protease (Capn10), cyclin-dependent kinase inhibitor 2a (Cdkn2a) for p16INK4a and p14ARF, and ubiquitin-associated proteins (Trp53inp1, Nbr1, Usp33). In addition, there were some novel interactions between AMPK and autophagy gene products across the two modules, indicated as “inbetween” in Table 4.

### 2.6. Testing for Molecular Functions and Cellular Components Enrichment by the Detected Modules

**Figure 4:**
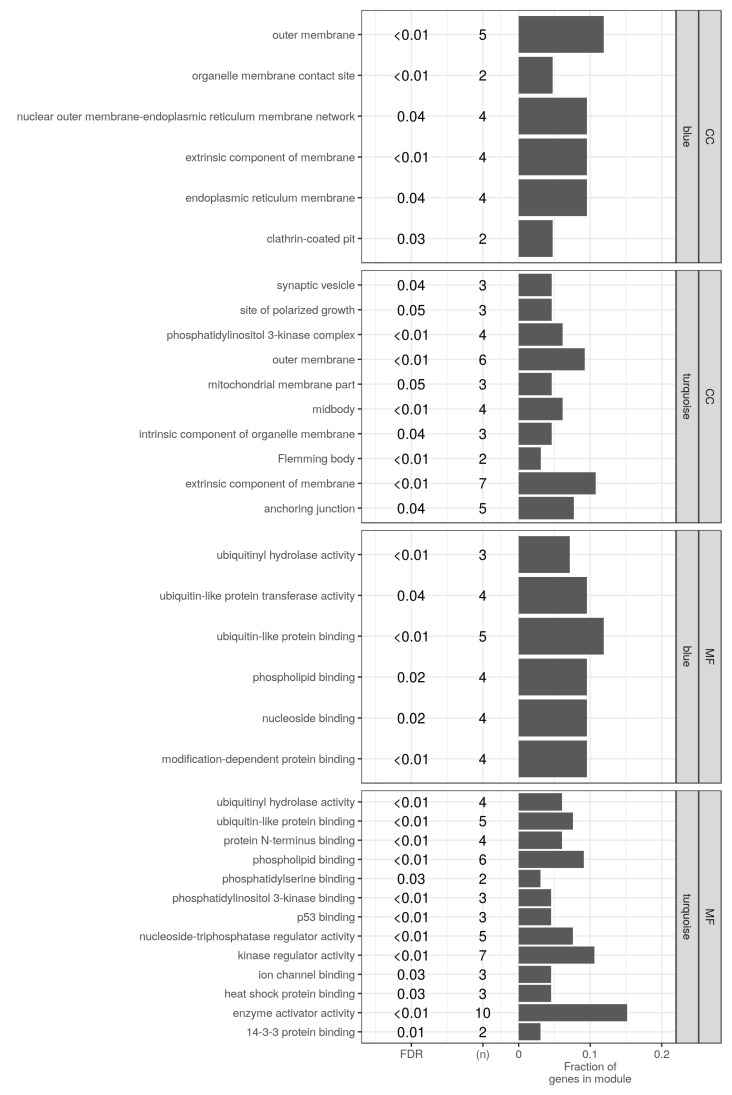
Enrichment of the gene ontology terms by the detected modules. The list of genes in the two detected modules (42, blue and 66, turquoise) were used to test for gene ontology terms enrichment. All terms in the Molecular Function (MF) and the Cellular component (CC) categories of the gene ontology were considered. Only significant terms at a false discovery rate (FDR) less than 0.1 are shown. For each term, the count (*n*) and the fractions of hits (bars) in the module are shown.

We used a list-based enrichment to specify the contributions of the modules to the differentiation process. The mouse gene ontology Molecular Function (MF) and Cellular Components (CC) terms were submitted to an enrichment analysis by the gene members of the detected modules (42 for blue and 66 for turquoise). The significant terms (FDR < 0.1) are shown in Figure 4 stratified by the category (MF/CC) and the module (blue/turquoise). As expected, the two modules share a number of MF terms, namely; ubiquitin-like protein binding, ubiquitinyl hydrolase activity and phospholipid binding. At the same time, several terms had significance by mutually exclusive enrichment by the modules. This includes the nucleoside binding and the ubiquitin-like protein transferase activity by the blue module; and a few protein kinase terms by the turquoise module. Similarly, two of the CC terms; extrinsic component of membrane and outer membrane were enriched by both modules, while others related to only one of the two modules.

Building on these two pieces of the analysis, the suggested novel AMPK interactions and the gene ontology enrichment by the members of the modules, we set out to specify the kind of functions that the AMPK-autophagy interactions were likely to be involved in. Table 5 shows how AMPK is functionally connected to autophagy gene products through several gene ontology terms such as membrane components, and regulation of kinases, enzymatic and ubiquitin activity. For examples, Prkab1 interacts with Sh3glb1, a Bax-interacting protein at the mitochondrial outer membrane and also with Trp53Inp2 for the uniquitin-like activity. In addition, Prkag1 associates with many autophagy gene products such as Usp33 for the uniquitin activity at the membrane anchoring junction, Cdkn2a for the regulation of kinase activity, and other cellular components for their molecular functions.

### 2.7. Preservation of AMPK-Autophagy Networks across Independent Datasets

**Figure 5:**
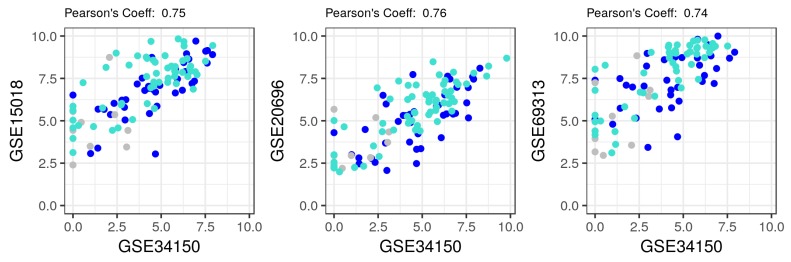
Average expression of AMPK and autophagy in multiple MDI-induced 3T3-L1 microarrays datasets. The log average expression values of AMPK and autophagy genes (*n* = 181) in the MDI-induced 3T3-L1 datasets (GSE15018, GSE20696 and GSE69313) are compared to the corresponding averages in the main dataset (GSE34150). Individual values are shown as colored points by their assigned modules. The Pearson’s correlation coefficient of the corresponding values is shown on top.

**Figure 6:**
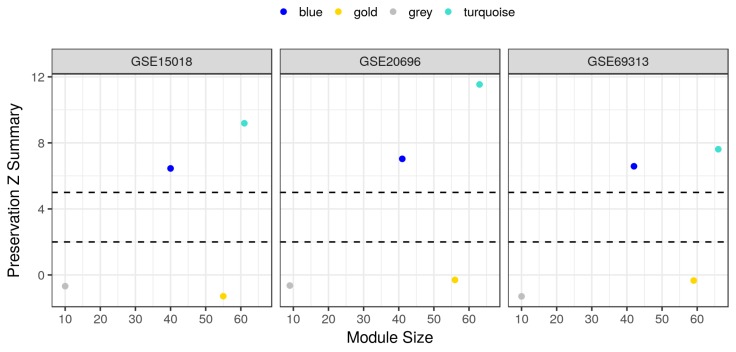
Module preservation *Z* summary across multiple MDI-induced 3T3-L1 microarrays datasets. The GSE34150 dataset was used to detect the highly co-expressed modules among AMPK and autophagy genes (42, blue; 66, turquoise; 10, gray, unassigned; and 55, gold , randomly assigned). The detected modules were used as a reference to calculate several preservation statistics in three independent datasets of similar design (GSE15018, GSE20696 and GSE69313). *Z* summary statistics and sizes of four modules are shown as colored points.

Finally, we validated these findings in three independent datasets of similar MDI-induced 3T3-L1 cells at different time points or differentiation stages. Three GEO microarray datasets (GSE15018, GSE20696 and GSE69313) were used to perform this step of the analysis (Table 1). The average log expression of the 181 genes of interest from the three datasets were first compared to these in the main dataset (Figure 5). As expected, the averages are highly correlated between the datasets (>0.74), a pre-requisite for the following module preservation analysis. A moderate to high preservation of the modules was observed in the three independent datasets (Figure 6). Generally, modules with a Z summary values between 5 and 10 are considered moderately preserved and these above 10 are considered highly preserved. In fact, the two modules: blue and turquoise, showed a summary statistics in that first category with at least 6 and 7, respectively, indicating that the interaction modules of the main dataset are well preserved in other independent datasets.

### 2.8. Validation of Selected Gene Products Correlations with Prkab1 and Prkag1

**Figure 7:**
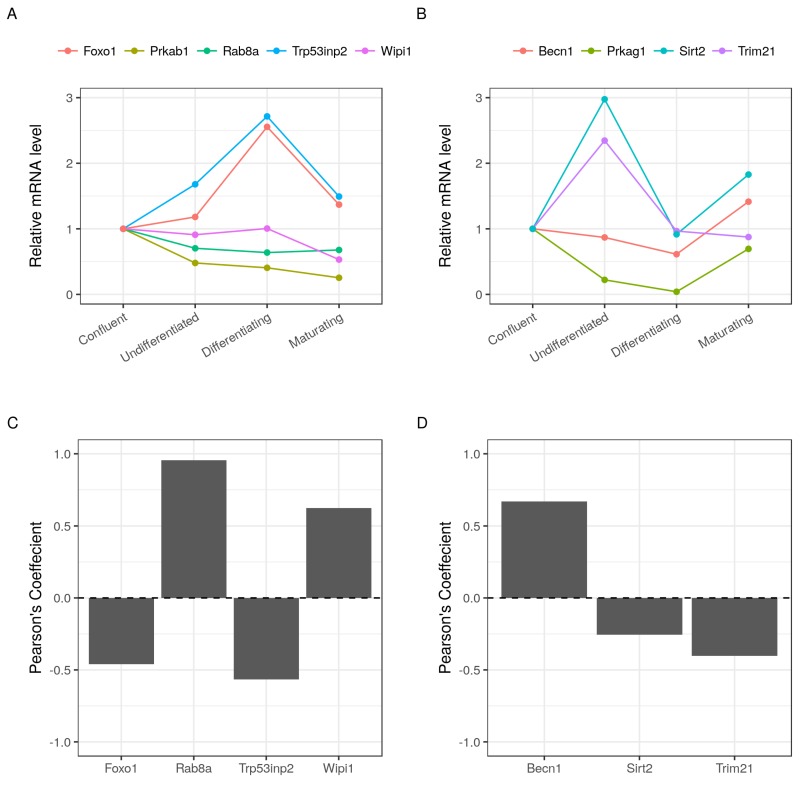
Validation of selected gene products expression and co-expression with AMPK subunits. Three independent samples of MDI-induced 3T3-L1 cells at four different time points corresponding to confluent, undifferentiated, differentiating and maturating stages were used to check the mRNA level of several gene products. (**A**,**B**) the $\Delta \Delta C_{t}$ values of five and four gene products, respectively, normalized by 18S and relative to the confluent cell stage are shown as points; (**C**,**D**) the Pearson’s coefficient of four and three gene products with Prkab1 and Prkag1, respectively, are shown as bars.

We selected several autophagy gene products that are highly correlated with the AMPK subunits for experimental validation using RT-qPCR (Figure 7A,B). The relative mRNA level of each group of genes were used to calculate the Pearson’s correlation coefficients with two AMPK subunits: Prkab1 and Prkag1. Although the resulting coefficient may vary, these calculated earlier due to the different sensitivities between microarrays and RT-qPCR, the directions of the correlation were the same as ones that we observed in the dataset (Figure 7C,D). In agreement with the suggested potential interaction of Prkab1 with Wipi1, Rab8a and Trp53inp2, strong correlations were validated. Prkag1 showed strong to moderate correlations with Becn1, Sirt2 and Trim21 as previously predicted by WGCNA.

## 3. Discussion

The adipocyte differentiation is a well regulated complex process. On one hand, this complexity allows for flexibility in response to different stimuli. For example, the over-expression of LC3 in 3T3-L1 pre-adipocytes produced a downstream activation of key regulators of adipogenesis and resulted in a differentiation pattern similar to that of the MDI induction [18]. On the other hand, this process likely involves a wide range of changes in transcription, translation and protein modification. In a previous study from our laboratory, we suggested that many autophagy genes were functionally associated with adipocyte differentiation using the RNA-Seq expression data [19]. We also showed that the mRNA level of key autophagy genes is specifically regulated at different time points, and clusters of these genes respond to the differentiation stimulus in a time-dependent manner. In particular, the subsets of organelle specific autophagy (e.g., mitophagy, reticulophagy, etc.) are highly regulated, suggesting a role in reorganizing the interacellulum and removing parts of the cell to adapt the morphological and metabolic changes of the mature adipocyte. Here, we explore the connection between autophagy and AMPK, which was established in conditions such as starvation, as it applies to the pre-adipocytes response to differentiation stimuli.

One aspect of this connection can be deduced from the observed positive correlation of the detected modules with the differentiation course (Figure 2A). In addition, the blue and the turquoise modules scores a significant (*p*-value < 0.001) protein–protein interaction (PPI) enrichment with an average clustering co-efficient of 0.4 and 0.5, respectively (STRING web interface). Although a detailed molecular link would be less clearer, AMPK gene products were evenly split among these modules, and had multiple edges with highly influential nodes (hubs) of well studied autophagy genes (Table 3 and Table 4). Thus, AMPK gene products are part of biologically connected autophagy modules, which seem to be fairly consequential in the process of adipocyte differentiation.

The AMPK complex is formed of one catalytic subunit (α) and two non-catalytic regulatory subunits (β and γ), each has more than one isoform encoded by a separate gene [20]. The different subunits contribute to the stability and activity of the complex, whereas the combinations of the different isoforms give rise to complexes that behave differently and/or are specific to certain tissues [21,22]. Probes corresponding to the genes that code the different isoforms of the subunits were consistently expressed at different levels. Moreover, they showed varying correlations with the cell differentiation stage (data not shown). We considered the subunits and the isoforms of the AMPK complex individually. *Prkab1* and *Prkag1* were expressed at higher levels, and they, therefore, are the main AMPK side of the reported interaction with the autophagy pathway (Table 4 and Table 5).

The adipocyte differentiation is characterized by events of increased lipogenesis and intracellular remodeling. AMPK is known for inhibiting the former and stimulating the latter. The absence of a reliable signal from the catalytic subunits of AMPK in our analysis may only enable a partial view. Nevertheless, we observe enrichment of ubiquitin activity and certain cellular component terms, probably akin to a form of localization, by autophagy genes co-expressed with the regulatory (β and γ) subunits of AMPK (Figure 4). One of these terms, ubiquitin-like protein binding, includes two gene products, *Trp53inp2* and *Nbr1*. Both are known to help the formation of autophagosome and the selective removal of ubiquitinated proteins through binding to LC3 [23,24,25]. Few other terms related to the organelle membranes appear interesting. Perhaps, the localization of AMPK at certain intracellular locations mediates the autophagy selectivity, as suggested before [26]. This is consistent with the description of two emerging mechanisms of AMPK regulation, namely by ubiquitination and sub-cellular distribution [27].

Together, it is possible that AMPK-autophagy connection is determined by the energy supply and demand of the differentiating cells. It is more likely that the two pathways interact dexterously with some temporospatial agility. For example, AMPK activates autophagy early in the differentiation course in response to the differentiation stimulus. In a later stage, autophagy might remove ubiquitinated AMPK to allow the accelerated fat accumulation. Finally, the localization of AMPK to certain intracellular organelles could guide their recycling or removal by selective autophagy. The implications of these features do not escape us, both AMPK and autophagy are involved in disorders such as obesity and diabetes [28,29]. The manipulation of one pathway could affect the outcomes controlled by the other. In addition, the timely intervention during adipocyte differentiation could preferentially favor certain consequences of the pathways’ interplay.

Nassiri and colleagues provided a system view of the adipogenesis and ranked the involved biological processes to identify the coordinated activity among them using the NASFinder method of publicly available omics data [30]. According to their analyses, the translational machinery, mitochondrial associated pathways, PPAR signaling, insulin and leptin signaling, and some membrane associated complexes are coordinately upregulated after adipocyte induction. Although they might be associated with AMPK or autophagy at the broad concept, they do not show any specific coordination between AMPK pathway and autophagy process. Here, we used the widely known WGCNA method to detect the conserved genetic networks of AMPK-autophagy gene products that might contribute to the process of differentiation [31,32]. Typically, it needs to input the list of differentially expressed genes among three or more experimental conditions [12]. In this study, we limited the analysis to the probes that mapped uniquely to AMPK and autophagy genes as defined in their gene ontology terms. The downside of limiting the analysis to a predefined set of genes is that the prospective findings would be limited to the available annotation, potential loss of signals from probes that map to genes not in the predefined gene set and the inclusion of probes that map to genes that are not actively changing among the conditions. On the other hand, this approach allows for simplifying the analysis steps and the interpretation of the results. The detected networks are more likely to have biologically meaningful consequences since they are formed of nodes that are known for certain functions in their pathways/gene sets. In addition, this allows for including genes that are highly correlated even though they don’t show the highest degree of differentiation among conditions. Certainly, some of these genes are involved in the biology of adipocyte differentiation either by maintaining essential cellular processes or they show subtle changes that wouldn’t be typically picked by the differential expression approach.

## 4. Materials and Methods

### 4.1. Data and Annotation Sources

#### 4.1.1. Gene Ontology

The Gene Ontology (GO) terms AMP-activated protein kinase (AMPK) (GO:0004679) and autophagy (GO:0006914) were used to identify the gene products (14 and 167, respectively) with known functions in the corresponding biological processes [33]. Similarly, GO was used to identify the molecular function (MF) and cellular component (CC) terms containing these gene products. GO was accessed through the GO.db and the mouse organism package org.Mm.eg.db [34,35].

#### 4.1.2. Microarrays Expression Data

To identify the relevant datasets, we queried the NCBI Gene Expression Omnibus (GEO) metadata by GEOmetadb [36]. The term ‘3T3-L1’ was used to search the titles of all entries, the query results were then searched manually and datasets of similar induction time-course design were included. The expression and the annotation data were then obtained using a GEOquery [37]. Table 1 summaries the four datasets that were used in this analysis. GSE34150 consists of 24 samples of MDI-induced 3T3-L1 pre-adipocytes at eight different time points corresponding to three differentiation stages (0 day, undifferentiated; two and four days, differentiating; 6–18 days, maturating).

#### 4.1.3. Protein–Protein Interactions

The STRING database was used to query all possible AMPK-autophagy protein–protein interactions that are reported with different evidence types [38]. The HUGO symbols of 181 genes were mapped to the ENSEMBL IDs before querying the database. STRINGdb was used to do the mapping, construct the query and obtain the results. The interactions were matched against the edges of the co-expression networks of the detected modules to label the edges with the type of evidence when they were previously reported.

### 4.2. Weighted-Gene Co-Expression Network Analysis

The package WGCNA was used to apply most of the necessary steps for weighted-gene co-expression network analysis on the GSE34150 dataset as described in the original publications [39]. Briefly, a co-expression measure (Pearson’s correlation coefficient) was calculated between each pair of genes. The coefficients were raised to the power of 5 to form an adjacency matrix. The adjacency matrix was then used to calculate the topological overlap similarity matrix (TOM). To detect modules and assign genes to them, a dissimilarity matrix is obtained (1 − TOM) and used as distances between genes. A hierarchical clustering was then performed and a gene tree is built. Upon cutting the tree at a certain height, genes nearby are assigned to modules, referred to as colors (names are arbitrarily assigned). The detected modules were then used to find the correlation with the phenotype and the preservation in independent datasets. To correlate the modules to the sample phenotypes or to each other, an eigengen or the principal components (PC) were calculated from the expression of their respective members and used as a representative summary. Finally, a module preservation analysis was performed by calculating various summary statistics on the detected modules in the test datasets [40].

### 4.3. Network Visualization and Analysis

The igraph package was used to visualize and analyze the detected modules [41]. The genes of interest were treated as nodes in a network graph and were connected by an edge if its weight—calculated from the TOM similarity between each pair of genes—passed a minimum threshold. Several graph statistics were used to determine the importance/centrality of genes and their interactions.

### 4.4. Gene Modules Over-Representation

The limma package was used to test for the over-representation of the detected modules in the GSE34150 dataset [42]. An index of the modules as gene sets, the expression data and comparison matrix based on the differentiation stage were used as input. A gene set is considered *over-represented* when it has a significantly higher fraction of differentially expressed genes than a randomly selected module of the same size. The clusterProfiler package was used to apply a similar list-based enrichment of GO terms by the detected modules [43]. Tests were adjusted for multiple testing using the False Discovery Rate (FDR) and a cutoff (0.1) was applied.

### 4.5. Cell Culture and RT-qPCR

3T3-L1 pre-adipocytes were cultured and induced for differentiation using MDI protocol as described before [19]. Total RNA was collected at four different time points corresponding the the major differentiation stages of the adipocytes (−2 day, full confluence; 0 day, undifferentiated; 10 h differentiating; and −6 day, maturating). The list of the primers that were used in the reaction are provided in (Appendix A). The $C_{t}$ values from the RT-qPCR reaction were normalized by a reference gene 18S and calibrated by the confluent samples ($\Delta \Delta C_{t}$) using the pcr R package [44].

### 4.6. Software Environment and Reproducibility

The data were obtained, processed and analyzed in an R environment and using multiple Bioconductor packages [45,46]. The full analysis was done and reproduced in an isolated environment based on docker (bioconductor/release_base2) [47]. The scripts for reproducing the analysis, figures and tables are available at https://github.com/MahShaaban/aacna. The instructions for reproducing the analysis are described in Appendix C.

## 5. Conclusions

In summary, we used the WGCNA to investigate the interactions of AMPK and autophagy gene products in the context of adipocyte differentiation. Two co-expression networks were found to be highly correlated with the time course of differentiation. We were able to validate the case of these networks in other independent datasets of similar experimental designs. These networks appear to be consequential in the response of the pre-adipocyte to the differentiation stimulus. Finally, we present several novel potential interactions between AMPK and autophagy gene products and link them to potential functions and cellular sites.

## Figures and Tables

**Table 1:** MDI-induced 3T3-L1 microarrays’ datasets.

Series ID	Platform ID	Samples	Included	(Contact, Year)	Reference
GSE15018	GPL6845	54	18	(Chin, 2009)	[14]
GSE20696	GPL1261	8	8	(Mikkelsen, 2010)	[15]
GSE34150	GPL6885	24	24	(Irmler, 2011)	[16]
GSE69313	GPL6246	48	12	(Renbin, 2015)	[17]

**Table 2:** AMPK and autophagy genes in different modules/colors.

Module/Color	AMPK	Autophagy
blue		*Acbd5*, *Atg5*, *Atm*, *Bmf*, *Bok*, *Casp1*, *Cln3*, *Dapk1*,
		*Dhrsx*, *Fbxl2*, *Fbxo7*, *Fis1*, *Hif1a*, *Lep*, *Map1lc3a*,
	*Prkaa2*, *Prkab1*,	*Mcl1*, *Mid2*, *Optn*, *Pik3c2a*, *Pink1*, *Prkaa2*, *Rab39b*,
	*Prkag2*, *Prkag3*, *Smok3b*	*Rab8a*, *Rragc*, *Sh3bp4*, *Sh3glb1*, *Sqstm1*, *Tbc1d5*, *Tnfaip3*,
		*Tpcn1*, *Tpcn2*, *Trim8*, *Trp53inp2*, *Vcp*,
		*Wdr45*, *Yod1*, *Zc3h12a*, *Zfyve1*
turquoise	*4921509C19Rik*, *Prkab2*, *Prkag1*	*Ager*, *Akt1*, *Bcl2*, *Becn1*, *Capn10*, *Cdkn2a*,
		*D17Wsu92e*, *Dap*, *Dcn*, *Depdc5*, *Ei24*, *Eif4g1*, *Eif4g2*,
		*Foxo1*, *Fundc1*, *Fundc2*, *Hmgb1*, *Hspa8*, *Htr2b*,
		*Ifng*, *Lamp2*, *Lars*, *Lmx1b*, *Lrrk2*, *Map1lc3b*, *Map2k1*,
		*Mapt*, *Mt3*, *Nbr1*, *Nlrp6*, *Pik3c3*, *Pik3r2*,
		*Pik3r4*, *Pim2*, *Plaa*, *Plekhf1*, *Plk2*, *Pycard*,
		*Rasip1*, *Rnf5*, *Rraga*, *Rragb*, *Sirt2*, *Smcr8*,
		*Smurf1*, *Stk11*, *Tcirg1*, *Tmem74*, *Trim21*, *Trp53inp1*,
		*Tsc2*, *Ubqln1*, *Ulk1*, *Usp10*, *Usp13*, *Usp30*,
		*Usp33*, *Vps4a*, *Vps4b*, *Wdr6*, *Wipi1*, *Wipi2*, *Xbp1*

**Table 3:** Top five hubs in the different module networks.

Module/Color	Gene	Degree	Betweenness	Closeness	Hub Score
blue	*Trp53inp2*	19	14.67	0.16	1
	*Map1lc3a*	19	22.71	0.16	0.99
	*Wdr45*	18	11.23	0.16	0.98
	*Pink1*	18	12.91	0.16	0.97
	*Dapk1*	18	14.48	0.16	0.96
turquoise	*Foxo1*	28	70.89	0.28	1
	*Dcn*	25	36.23	0.28	0.96
	*Xbp1*	24	29.59	0.28	0.93
	*Plk2*	24	60.28	0.27	0.9
	*Eif4g1*	24	41.01	0.27	0.9

**Table 4:** Summary of reported and novel AMPK-autophagy interactions.

Module/Color	AMPK	Autophagy
blue	*Prkab1*	*Bmf*, *Dapk1*, *Rab8a*, *Sh3glb1*, *Trp53inp2*
	*Prkag3*	*Rragc* ${}^{4}$
inbetween	*Prkab1*	*Tsc2*${}^{2,4}$, *Ubqln1*, *Wipi1*
	*Prkab2*	*Zc3h12a*
	*Prkag3*	*Usp33*
turquoise	*Prkag1*	*Akt1*${}^{1,3,4}$, *Bcl2*, *Becn1*, *Capn10*, *Cdkn2a*, *Dcn*${}^{3,4}$, *Eif4g1*, *Foxo1*${}^{2}$,
		*Fundc1*, *Lamp2*, *Lars*, *Map1lc3b*, *Nbr1*, *Plk2*, *Sirt2*${}^{3,4}$, *Trim21*,
		*Trp53inp1*, *Usp33*, *Vps4a*, *Wipi2*${}^{3,4}$, *Xbp1*

${}^{1}$ Coexpression in the same or in other species (transferred by homology). ${}^{2}$ Database gathered from curated databases. ${}^{3}$ Experiments gathered from other protein–protein interaction databases. ${}^{4}$ Textmining extracted from the abstracts of scientific literature.

**Table 5:** AMPK and autophagy interactions by gene ontology term.

Module/Color	Ontology	AMPK	Term	Autophgy
blue	CC	*Prkab1*	outer membrane	*Sh3glb1*
	MF		nucleoside binding	*Dapk1*, *Rab8a*
			ubiquitin-like protein binding	*Trp53inp2*
		*Prkag3*	nucleoside binding	*Rragc*
turquoise	CC	*Prkag1*	anchoring junction	*Usp33*
			extrinsic component of membrane	*Becn1*, *Wipi2*
			Flemming body	*Vps4a*
			intrinsic component of organelle membrane	*Fundc1*, *Lamp2*
			midbody	*Sirt2*, *Vps4a*
			mitochondrial membrane part	*Fundc1*
			outer membrane	*Bcl2*, *Capn10*, *Fundc1*
			phosphatidylinositol 3-kinase complex	*Becn1*
	MF		14-3-3 protein binding	*Akt1*
			enzyme activator activity	*Lars*
			kinase regulator activity	*Cdkn2a*, *Dcn*
			nucleoside-triphosphatase regulator activity	*Lars*
			p53 binding	*Cdkn2a*
			phosphatidylinositol 3-kinase binding	*Becn1*, *Xbp1*
			phospholipid binding	*Akt1*, *Wipi2*
			protein N-terminus binding	*Cdkn2a*, *Dcn*
			ubiquitin-like protein binding	*Nbr1*, *Sirt2*
			ubiquitinyl hydrolase activity	*Usp33*

**Figure A1:**
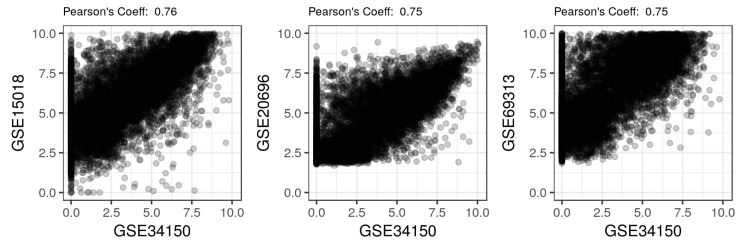
Average expression of all probes in multiple MDI-induced 3T3-L1 microarrays datasets. The log average expression values of all probes in the MDI-induced 3T3-L1 datasets (GSE15018, GSE20696 and GSE69313) are compared to the corresponding averages in the main dataset (GSE34150). Individual values are shown as points. The Pearson’s correlation coefficient of the corresponding values is shown on top.

**Figure A2:**
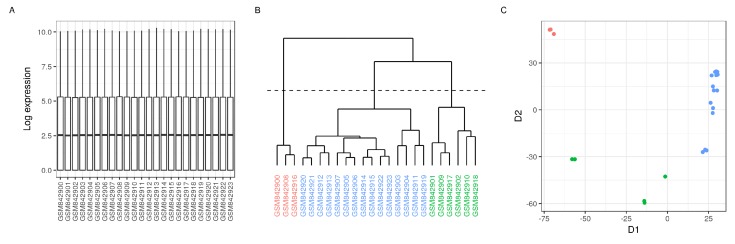
Quality assessment and exploration of the main microarrays dataset. Twenty-four samples of MDI-treated 3T3-L1 cells of the microarray series (GSE34150) were obtained from GEO along with the corresponding annotation data (GPL6885); (**A**) the distribution of the log expression of (*n* = 25,697) probes from all samples as box plots; (**B**) hierarchical clustering based on the euclidean distances of all samples; (**C**) multi-dimensional scaling (MDS) of all samples. Colors represent the cell stage/time point (green, undifferentiated; dark green, differentiating and red, maturating).

**Figure A3:**
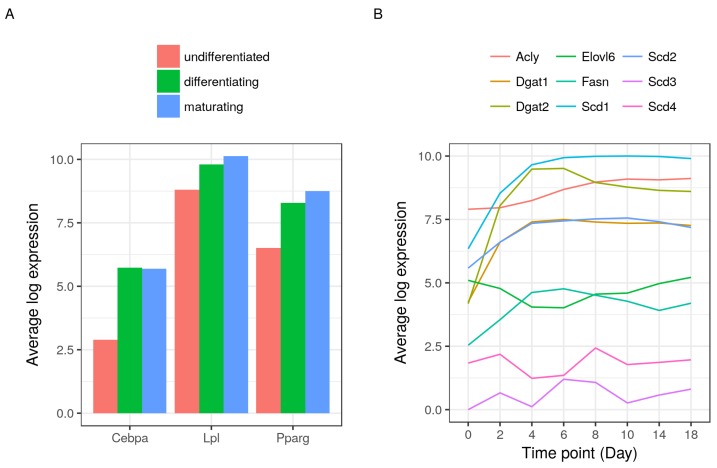
Differentiation and lipogensis markers in differentiating adipocytes. Average log expression values from 24 samples of MDI-induced 3T3-L1 cells (GSE34150) at 3 differentiation stages and 8 time points (0 day, undifferentiated (red); 2 and 4 days, differentiating (green); 6–18 days, maturating (blue)) from (**A**) differentiation markers and (**B**) lipogenesis markers are shown as bars and lines, respectively. *Cebpa*, CCAAT/enhancer binding protein (C/EBP), alpha; *Lpl*, lipoprotein lipase; *Pparg*, peroxisome proliferator activated receptor gamma; *Acly*, ATP Citrate Lyase; *Dgat*, Diacylglycerol *O*-Acyltransferase; *Elov6*, Fatty Acid Elongase 6; *Fasn*, Fatty Acid Synthase; *Scd*, Stearoyl-CoA Desaturase.

**Figure A4:**
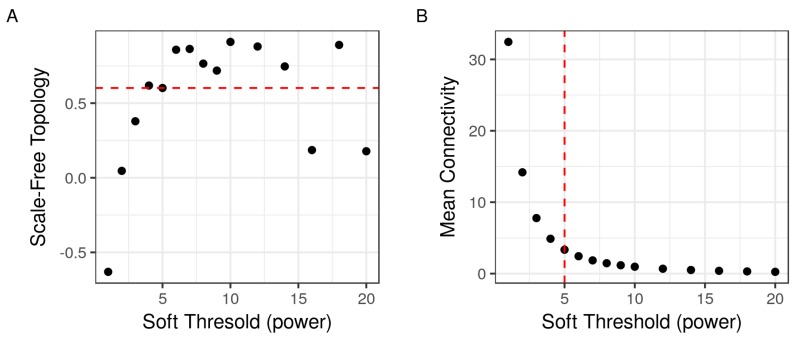
Scale free topology for multiple power values. Expression data from 24 samples (GSE34150) MDI-induced 3T3-L1 at different time points were used to calculate an (*n* × *n*) similarity matrix (*n* = 181 genes), which were used to obtain the weighed networks by raising them to multiple power values. For each value, a scale free topology index was calculated. (**A**) the fit indices, the slopes multiplied by the *R* squared $R^{2}$ values, are shown for each power value as points; (**B**) the mean connectivity, average edges shared by a node, for the resultant network at each power value are shown as points. Red lines represent the choice of power that satisfy both high *R* squared $R^{2}$ values and high connectivity.

**Figure A5:**
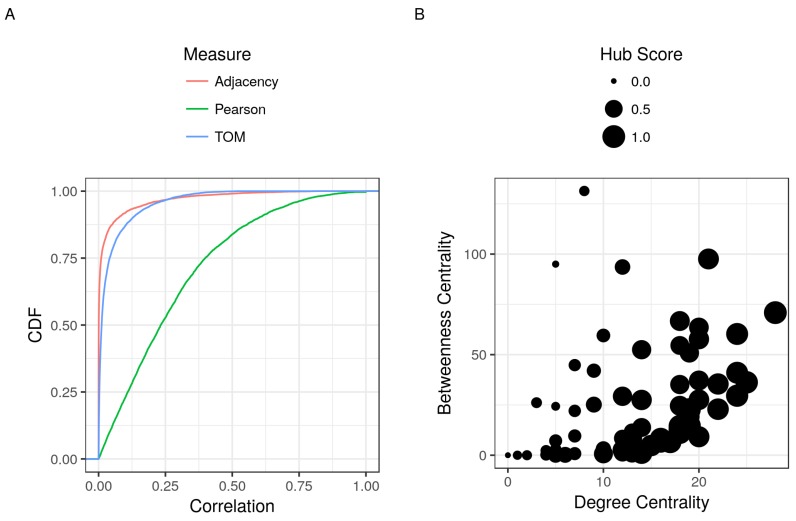
Gene similarity and node centrality measures. (**A**) the cumulative distribution function (CDF) of three correlation/similarity measures of AMPK and autophagy genes (*n* = 181) are shown as colored lines (green, Pearson’s correlation coefficients; red, adjacency; and blue, TOM); (**B**) three centrality measures for all nodes in the two detected modules are as points. The degree centrality on the *x*-axis, the betweenness centrality on the *y*-axis and the hub score as the point size.

**Figure A6:**
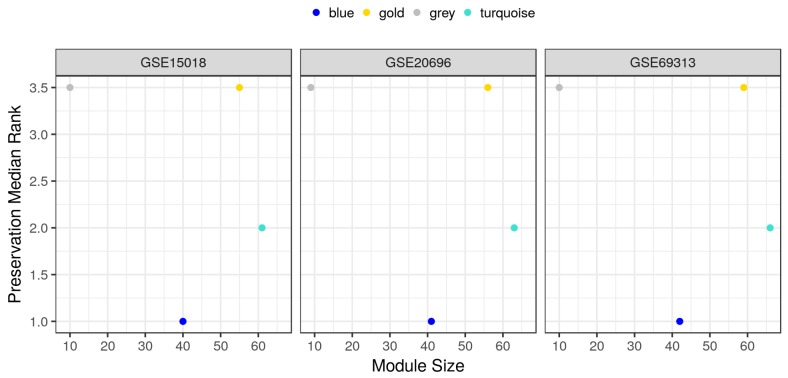
Module preservation ranks across multiple MDI-induced 3T3-L1 microarrays datasets. The GSE34150 dataset was used to detect the highly co-expressed modules among AMPK and autophagy genes (42, blue; 66, turquoise; 10, gray, unassigned; and 55, gold, randomly assigned). The detected modules were used as a reference to calculate several preservation statistics in three independent datasets of similar design (GSE15018, GSE20696 and GSE69313). The median ranks of the preservation statistics and the sizes of four modules are shown as colored points.

**Figure A7:**
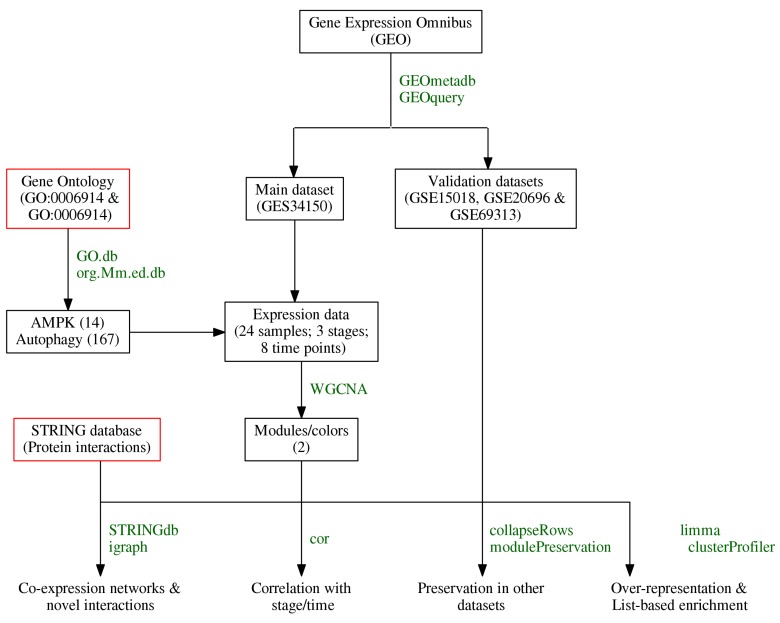
Workflow of the study.

## Abbreviations

AMPK	AMP-Activated Protein Kinase
CC	Cellular Components
FDR	False Discovery Rate
GEO	Gene Expression Omnibus
GO	Gene Ontology
MDS	Multi-Dimensional Scaling
mTOR	Mechanistic Target Of Rapamycin Kinase
MF	Molecular Function
PC	Principal Component
PPI	Protein–Protein Interaction
RT-qPCR	Real-Time Quantitative Polymerase Chain Reaction
TOM	Topological Overlap Matrix
ULK1	Unc-51 Like Autophagy Activating Kinase 1
WGCNA	Weighted Gene Co-expression Network Analysis

## Appendix A. Datasets and Annotations

This appendix contains details of the datasets and the gene annotation used in the study.

### Appendix A.1. Time Point of Samples in the Microarrays Datasets

We queried the metadata of the Gene Expression omnibus (GEO) for microarrays datasets of MDI-induced 3T3-L1 pre-adipocytes at different time points that covers the various differentiation stages. The datasets were then manually checked for containing sufficient phenotype and annotation data. A few datasets were generated using custom microarrays chips of a few thousand probes and were excluded for not containing a sufficient number of the probes of interest. In Table A1, we listed four datasets included in this study and the time points (in hours) of their samples.

**Table A1:** Sample time points in the different datasets.

Time Point (hours)	GSE15018	GSE20696	GSE34150	GSE69313
−48		2		
0		2	3	3
1	1			
2	1			
3	1			
4	1			
5	1			
6	1			3
7	1			
8	1			
9	1			
10	1			
11	1			
12	1			
13	1			
14	1			
15	1			
16	1			
17	1			
18	1			
24				3
48		2	3	
72				3
96			3	
144			3	
168		2		
192			3	
240			3	
336			3	
432			3	

### Appendix A.2. Comparing the Average Expression in Four Datasets

To ensure that the different datasets are exhibiting comparable probe expression, we compared the average expression of all common probes in three datasets (GSE15018, GSE20696 and GSE69313) with the main (GSE34150) dataset (Figure A1).

### Appendix A.3. Data Quality Assessment

The accession number (GSE34150) was used to obtain the expression matrix and the metadata of the dataset. Several quality assessment measures were applied to ensure the suitability of the data for the downstream analysis (Figure A2).

### Appendix A.4. Confirming Differentiation and Lipogenesis

Several adipogenic and lipogenic of markers transcriptional changes are expected during the course of the 3T3-L1 cell differentiation. Figure A3 show the log expression level of some of these markers at different stages in the GSE34150 dataset.

### Appendix A.5. RT-qPCR Primer Sequences

The following nine primers were used to validate the expression and correlations of the corresponding genes products with the subunits of AMPK during the time course of 3T3-L1 differentiation (Table A2).

**Table A2:** RT-qPCR primer sequences.

Name	Forward (5${}^{\prime }$ to 3${}^{\prime }$)	Reverse (3${}^{\prime }$ to 5${}^{\prime }$)
*18S*	ACCGCAGCTAGGAATAATGGA	GCCTCAGTTCCGAAAACCA
*Becn1*	CAGGAACTCACAGCTCCATTAC	CCATCCTGGCGAGTTTCAATA
*Prkab1*	GAGATCAAGGCTCCAGAGAAAG	GTTGAAGGACCCAGACAAGTAG
*Prkag1*	GAACTGGAGGAGCACAAGATAG	GGGAGCCTGTGGATCTTATTT
*Rab8a*	GCTCGATGGCAAGAGGATTA	CTGTAGTAGGCTGTCGTGATTG
*Sirt2*	CATAGCCTCTAACCACCATAGC	GTAGCCTGTTGTCTGGGAATAA
*Trim21*	GATAGCCCAGAATACCAAGAAGAG	GCCCATCTTCCTCACAGAATAG
*Trp53inp2*	GGTGAAGCGCTGGAACAT	CACAACTACCTCAGCGCAGC
*Wipi1*	GTGTGTCTAGACGACGAGAATG	GACTTCTGAGGTAGGCTTCTTG

### Appendix A.6. Gene Ontology Annotation

To define the sets of genes involved in the AMPK and autophagy pathways, we turned to the gene ontology (GO) annotations. GO identifies the AMP-dependent protein kinase activity (GO:0004679) as catalysis of the reaction: ATP + a protein = ADP + a phosphoprotein. In total, 14 genes were identified to be involved in the pathway and its regulation and are referred to as AMPK genes in the manuscript. Similarly, GO defines the term autophagy (GO:0006914) as the catabolic process in which the cells digest parts of their own cytoplasm. It contains 10 children/subcategory terms and 167 genes. The gene symbols of AMPK and autophagy gene sets are listed in Table A3.

**Table A3:** Gene members of the AMPK and autopahgy gene ontology terms.

Category	Term	Genes
AMPK	AMP-activated protein kinase activity	*Prkab1*, *Prkag1*, *Smok1*, *Smok2a*, *Prkaa1*, *Prkaa2*, *Prkab2*, *Prkag2*,
		*Smok2b*, *Prkag3*, *4921509C19Rik*, *Smok3a*, *Smok3b*, *Smok3c*
autophagy	autophagy of mitochondrion	*Atg5*, *Rb1cc1*, *Cdkn2a*, *Capn10*, *Park2*, *Wipi1*, *Wdr45*, *Becn1*,
		*Fis1*, *Atg4b*, *Map1lc3a*, *Wdr45b*, *Cisd2*, *Fundc2*, *Map1lc3b*, *Atg12*, *Atg3*,
		*Pink1*, *Fbxo7*, *Fundc1*, *Atg7*, *Wipi2*, *Atg2b*, *Usp30*, *Atg9b*,
		*Ambra1*, *Atg4d*, *Atg4c*, *Atg9a*, *Atg2a*, *Atg4a*
	autophagy of nucleus	*Atg5*, *Wipi1*, *Wdr45*, *Becn1*, *Atg4b*, *Wdr45b*, *Atg12*, *Atg3*,
		*Wipi2*, *Trappc8*, *Atg2b*, *Becn2*, *Atg4d*, *Atg4c*, *Atg2a*, *Atg4a*
	autophagy of peroxisome	*Rb1cc1*, *Acbd5*, *Pik3r4*, *Trappc8*, *Pik3c3*
	chaperone-mediated autophagy	*Hspa8*, *Lamp2*
	late endosomal microautophagy	*Hspa8*, *Vps4b*, *Vps4a*
	macroautophagy	*Atg5*, *Cln3*, *Ei24*, *Nbr1*, *Sqstm1*, *Pik3c2a*, *Plaa*, *Ulk1*,
		*Tcirg1*, *Ubqln1*, *Becn1*, *Ubxn6*, *Map1lc3a*, *Map1lc3b*, *Atg3*,
		*Trp53inp2*, *Tbc1d5*, *Atg7*, *Pik3r4*, *Zfyve1*, *D17Wsu92e*, *Pik3c3*, *Yod1*,
		*Tmem74*, *Pik3c2b*, *Vcp*, *Atg14*
	negative regulation of autophagy	*Akt1*, *Bcl2*, *Eif4g2*, *Htr2b*, *Il3*, *Lep*, *Lepr*, *Mcl1*,
		*Mt3*, *Ptpn22*, *Tnfaip3*, *Rnf5*, *Mtor*, *Sirt2*, *Rraga*, *Washc1*,
		*Rasip1*, *Zkscan3*, *Dapl1*, *Wdr6*, *Tbc1d14*, *Lars*, *Bmf*, *Eif4g1*,
		*Dap*, *Kdm4a*, *Herc1*, *Rubcn*
	positive regulation of autophagy	*Ager*, *Dcn*, *Hif1a*, *Ifng*, *Pim2*, *Prkd1*, *Plk2*, *Trim21*,
		*Stk11*, *Tfeb*, *Tsc2*, *Xbp1*, *Mid2*, *Map2k1*, *Irgm2*, *Mefv*,
		*Sh3glb1*, *Nprl2*, *Becn1*, *Tmem59*, *Foxo1*, *Trp53inp1*, *Lrrk2*, *Mtdh*,
		*Trp53inp2*, *Dapk1*, *Optn*, *Plekhf1*, *Atg7*, *Svip*, *Uvrag*, *Tlr9*,
		*Trim8*, *Sh3bp4*, *Prkaa1*, *Ticam1*, *Prkaa2*, *Flcn*, *Ambra1*, *Zc3h12a*,
		*Dhrsx*, *Tpcn1*, *Rnf152*, *Trim65*, *Atg14*
	protein targeting to vacuole involved in autophagy	*Smurf1*
	regulation of autophagy	*Atm*, *Bcl2*, *Casp1*, *Hmgb1*, *Lmx1b*, *Rab8a*, *Mapt*, *Pik3r2*, *Pip4k2a*, *Usp10*,
		*Xbp1*, *Park2*, *Bok*, *Rragd*, *Rragc*, *Iigp1*, *Trp53inp1*, *Lrrk2*, *Pycard*, *Cisd2*,
		*Dram2*, *Soga3*, *Kat8*, *Rab39b*, *Rraga*, *Mtcl1*, *Dram1*, *Mfsd8*, *Fbxl2*, *Usp13*,
		*3110043O21Rik*, *Rptor*, *Chmp4b*, *Nlrp6*, *Pip4k2b*, *Pip4k2c*, *Usp33*, *Wdr41*,
		*Tpcn2*, *Smcr8*, *Lamp3*, *Rragb*, *Wdr24*, *Depdc5*, *Soga1*, *Fbxw7as1*

## Appendix B. Rational of Analysis Directive

This appendix contains a brief discussion of some of the decision that were made at different steps of the analysis and the rationals behind them. Specifically, we describe the steps of constructing the co-expression networks and the centrality measures that were applied to them.

### Appendix B.1. Choosing the Network Power Threshold

A critical choice in constructing the co-expression networks is setting the soft threshold (power) to which the adjacency matrix is raised. A Scale Free Topology (SFT) measure is calculated by multiplying the a slope and a fitted R square and a mean connectivity of the networks at different power values (Figure A4). A power value of 5 was chosen to satisfy the SFT the most.

### Appendix B.2. Steps of Constructing the Weighed Co-Expression Networks

In this section, we discuss the different steps for calculating the similarity measures that was used in constructing the networks as well as comparing to the intermediary forms in representing the notation of co-expression. Particularly, the issue of penalizing the low correlation values. Three main steps are necessary:

- The absolute values of either Pearson’s (default) or Spearman’s coefficient can be used to provide an initial similarity measure $s_{ij}$ between each pair of nodes (ij) as in: $s_{ij}=|cor\left(ij\right)|$.
- The similarity matrix is then transformed to and adjacency matrix by elevating it to a selected power β as in: $a_{ij}=s_{ij}^{\beta }$.
- This matrix is then used to calculate the connectivity/weight of each pair of nodes as follows:
  $$w_{ij}=\frac{l_{ij}+a_{ij}}{min\left\{k_{i}+k_{j}\right\}+1-a_{ij}},$$
  where $l_{ij}=\sum_{u}a_{iu}a_{uj}$ and $k_{i}=\sum_{u}a_{iu}$. These weights (also not shown) are finally used to calculate a dissimilarity measure for clustering and detecting the gene modules as in: $d_{ij}^{w}=1-w_{ij}$.

Figure A5A shows the cumulative distribution functions of the final TOM similarity measure and the two intermediaries; Pearson’s correlation and the adjacency (after being raised to the power of 5).

### Appendix B.3. Node Centrality Measures

To determine the importance of each node/gene in the networks, we relied on multiple measures of centrality or node influence. These measures are calculated as follows:

- Degree Centrality: the number of edges/connections shared by a node. The Degree of a node *v* is given by:
  $$Degree\left(v\right)=\sum_{j}a_{v,j},$$
  where $a_{v,j}$ is the adjacency matrix of the network or the sum of the corresponding row.
- Betweenness Centrality: The number of the occasion of a node falls on the shortest between two other nodes. The Betweenness of a node *v* is given by:
  $$Betweenness\left(v\right)=\sum_{s\neq v\neq t\in V}\frac{\sigma_{st}\left(v\right)}{\sigma_{st}}$$
  or the fraction of the shortest paths between each pair of nodes (s,t) that passes through *v*.
- Hub Score/Eigenvector: a measure of the influence of a given node in a network. The Eigenvector of a node *v* is given by:
  $$Eigenvector\left(v\right)=\frac{1}{\lambda }\sum_{t\in M(v)}x_{t}=\frac{1}{\lambda }\sum_{t\in G}a_{vt}x_{t}$$
  or the sum of the scores $x_{i}$ of its neighboring nodes M(v) in the network *G*.

Figure A5B shows the different centrality measures and the correlations between them. Specifically, we found that the degree centrality is highly correlated with the hub scores (Pearson’s coefficient about 0.73) and less so with the betweenness centrality (Pearson’s coefficient about 0.44).

### Appendix B.4. Network Preservation

The authors of the WGCNA method suggests using composite preservation summaries to evaluate the evidence of the preservation of the detected modules in the test dataset/s as opposed to using individual statistics as they measure different aspect of the preservation. In the main text, we showed the $Z_{summary}$ for the preservation of the two detected modules in three test datasets. Here, we briefly expand on what this statistics composed of and show additional statistics supporting the preservation of the modules.

The $Z_{summary}$ statistics is given by:

$$Z_{summary}=\frac{Z_{connectivity}+Z_{density}}{2}.$$

Each of the two Z summaries are further composed of several statistics, the details of which are provided in the references.

As opposed to the $Z_{summary}$ as evidence for module preservation, the median rank of the preservation is more robust to the module sizes. However, it is more informative in comparing preservation of modules relative to each other, as it is based on the ranks of the observed preservation statistics of the module. The lower the median rank of the module, the more preservation it exhibits in the test set. Figure A6 shows the median ranks/relative preservation of the modules with their sizes.

## Appendix C. A Note on Reproducing the Analysis

This is a detailed description of the details required to reproduce the analysis from the source code. We first introduce the way to obtain the source code, the software environment and the commands to run the analysis script and generate the figures and tables that appear in this manuscript. In Figure A7, we show the workflow of the study listing the steps, datasets and software packages that were used in the analysis.

### Appendix C.1. Setting up the Docker Environment

The analysis was run on a docker image based on the the latest bioconductor/release_base2. Other R packages were added to the image and were made available as an image that can be obtained and launched on any local machine running docker:

$ docker pull mahshaaban/analysis_containers:bioc_wgcna,
$ docker run -it mahshaaban/analysis_containers:bioc_wgcna bash.

### Appendix C.2. Obtaining the Source Code

The source code is hosted publicly on a repository on github in a form of research compendium. This includes the functions used throughout the analysis as an R package, the scripts to run the analysis and finally the scripts to reproduce the figures and tables in this manuscript. From within the container, git can be used to cloned the source code. The cloned repository contains a sub-folder called ’analysis/scripts’, which can be used to reproduce the analysis from scratch:

- 01.analysis.R This script loads the required libraries, download the data and run all the steps of the analysis described in the manuscript,
- figures/A sub-folder with a separate file for each graph in the manuscript,
- tables/A sub-folder with a sepearte file for each table in the manuscript.

The following code clones the repository containing the source code:

$ git clone http://github.com/MahShaaban/aacna.

### Appendix C.3. Running the Analysis

The analysis scripts is organized to be ran using a single ’make’ command. This will first load the necessary functions and run the main analysis and save the data in an R object ’wgcna.rda’. This will be used to generate the figures and graphs. In addition, a log file is generated in the sub-folder ’log/’ for each script that can be used for troubleshooting.

To do that, the ’make’ command should be invoked from withing the ’analysis/’ sub-folder.

$ **cd** aacna/analysis/
$ make

### Appendix C.4. Details of the R Environment

The version of R that was used to perform this analysis is the 3.4.2 (28 September 2017) on x86_64-pc-linux-gnu. The ’DESCRIPTION’ file in the main repository contains further details about the dependencies and the license of this work.

## References

[1] Zhang Y., Goldman S., Baerga R., Zhao Y., Komatsu M., Jin S. (2009). Adipose-specific deletion of autophagy-related gene 7 (*atg7*) in mice reveals a role in adipogenesis. Proc. Natl. Acad. Sci. USA.

[2] Baerga R., Zhang Y., Chen P.H., Goldman S., Jin S. (2009). Targeted deletion of autophagy-related 5 *(atg5*) impairs adipogenesis in a cellular model and in mice. Autophagy.

[3] Singh R., Xiang Y., Wang Y., Baikati K., Cuervo A.M., Luu Y.K., Tang Y., Pessin J.E., Schwartz G.J., Czaja M.J. (2009). Autophagy regulates adipose mass and differentiation in mice. J. Clin. Investig..

[4] Singh R., Kaushik S., Wang Y., Xiang Y., Novak I., Komatsu M., Tanaka K., Cuervo A.M., Czaja M.J. (2009). Autophagy regulates lipid metabolism. Nature.

[5] Gwinn D.M., Shackelford D.B., Egan D.F., Mihaylova M.M., Mery A., Vasquez D.S., Turk B.E., Shaw R.J. (2008). AMPK Phosphorylation of raptor mediates a metabolic checkpoint. Mol. Cell.

[6] Inoki K., Zhu T., Guan K.L. (2003). TSC2 Mediates Cellular Energy Response to Control Cell Growth and Survival. Cell.

[7] Kim J., Kundu M., Viollet B., Guan K.L. (2011). AMPK and mTOR regulate autophagy through direct phosphorylation of Ulk1. Nat. Cell Biol..

[8] Egan D.F., Shackelford D.B., Mihaylova M.M., Gelino S., Kohnz R.A., Mair W., Vasquez D.S., Joshi A., Gwinn D.M., Taylor R. (2011). Phosphorylation of ULK1 (hATG1) by AMP-activated protein kinase connects energy sensing to mitophagy. Science.

[9] Green H., Kehinde O. (1975). An established preadipose cell line and its differentiation in culture II. Factors affecting the adipose conversion. Cell.

[10] Ntambi J.M., Young-Cheul K. (2000). Adipocyte differentiation and gene expression. J. Nutr..

[11] Roberts R., Hodson L., Dennis A.L., Neville M.J., Humphreys S.M., Harnden K.E., Micklem K.J., Frayn K.N. (2009). Markers of de novo lipogenesis in adipose tissue: Associations with small adipocytes and insulin sensitivity in humans. Diabetologia.

[12] Zhang B., Horvath S. (2005). A General Framework for Weighted Gene Co-Expression Network Analysis. Stat. Appl. Genet. Mol. Biol..

[13] Edgar R., Domrachev M., Lash A.E. (2002). Gene Expression Omnibus: NCBI gene expression and hybridization array data repository. Nucleic Acids Res..

[14] Chin K. (2010). Dataset: A Time Course Analysis of the Effects of Prieurianin in the Mouse Preadipocytes 3T3-L1 Cells.

[15] Mikkelsen T.S., Xu Z., Zhang X., Wang L., Gimble J.M., Lander E.S., Rosen E.D. (2010). Comparative epigenomic analysis of murine and human adipogenesis. Cell.

[16] Horsch M., Beckers J., Adamski J., Halama A. (2015). Dataset: Genome-Wide Expression Profiling Analysis of a Time Course of Differentiating Adipocytes.

[17] Zhang M., Zhang Y., Ma J., Guo F., Cao Q., Zhang Y., Zhou B., Chai J., Zhao W., Zhao R. (2015). Dataset: Effect of siRNA Knock-Down of FTO on 3T3-L1 Cell Differentiation.

[18] Hahm J.R., Ahmed M., Kim D.R. (2016). RKIP phosphorylation–dependent ERK1 activation stimulates adipogenic lipid accumulation in 3T3-L1 preadipocytes overexpressing LC3. Biochem. Biophys. Res. Commun..

[19] Ahmed M., Quoc Nguyen H., Seok Hwang J., Zada S., Huyen Lai T., Soo Kang S., Ryong Kim D. (2018). Systematic characterization of autophagy-related genes during the adipocyte differentiation using public-access data. Oncotarget.

[20] Davies S.P., Hawley S.A., Woods A., Carling D., Haystead T.A., Hardie D.G. (1994). Purification of the AMP-activated protein kinase on ATP-*γ*-sepharose and analysis of its subunit structure. Eur. J. Biochem..

[21] Dasgupta B., Chhipa R.R. (2016). Evolving lessons on the complex role of AMPK in normal physiology and cancer. Trends Pharmacol. Sci..

[22] Ross F.A., Jensen T.E., Hardie D.G. (2016). Differential regulation by AMP and ADP of AMPK complexes containing different subunit isoforms. Biochem. J..

[23] Mauvezin C., Orpinell M., Francis V.A., Mansilla F., Duran J., Ribas V., Palacín M., Boya P., Teleman A.A., Zorzano A. (2010). The Nuclear Cofactor DOR Regulates Autophagy in Mammalian and Drosophila Cells.

[24] Sala D., Ivanova S., Plana N., Ribas V., Duran J., Bach D., Turkseven S., Laville M., Vidal H., Karczewska-Kupczewska M. (2014). Autophagy-regulating TP53INP2 mediates muscle wasting and is repressed in diabetes. J. Clin. Investig..

[25] Kirkin V., Lamark T., Sou Y.S., Bjørkøy G., Nunn J.L., Bruun J.A., Shvets E., McEwan D.G., Clausen T.H., Wild P. (2009). A Role for NBR1 in Autophagosomal Degradation of Ubiquitinated Substrates. Mol. Cell.

[26] Liang J., Xu Z.X., Ding Z., Lu Y., Yu Q., Werle K.D., Zhou G., Park Y.Y., Peng G., Gambello M.J. (2015). Myristoylation confers noncanonical AMPK functions in autophagy selectivity and mitochondrial surveillance. Nat. Commun..

[27] Jeon S.M. (2016). Regulation and function of AMPK in physiology and diseases. Exp. Mol. Med..

[28] Kola B., Grossman A., Korbonits M. (2008). The role of AMP-activated protein kinase in obesity. Front. Horm Res..

[29] Goldman S., Zhang Y., Jin S. (2010). Autophagy and adipogenesis: implications in obesity and type II diabetes. Autophagy.

[30] Nassiri I., Lombardo R., Lauria M., Morine M.J., Moyseos P., Varma V., Nolen G.T., Knox B., Sloper D., Kaput J. (2016). Systems view of adipogenesis via novel omics-driven and tissue-specific activity scoring of network functional modules. Sci. Rep..

[31] Stuart J.M., Segal E., Koller D., Kim S.K. (2003). A gene-coexpression network for global discovery of conserved genetic modules. Science.

[32] Carter S.L., Brechbühler C.M., Griffin M., Bond A.T. (2004). Gene co-expression network topology provides a framework for molecular characterization of cellular state. Bioinformatics (Oxford England).

[33] Ashburner M., Ball C.A., Blake J.A., Botstein D., Butler H., Cherry J.M., Davis A.P., Dolinski K., Dwight S.S., Eppig J.T. (2000). Gene Ontology: Tool for the unification of biology. Nat. Genet..

[34] Carlson M. (2016). org.Mm.eg.db: Genome Wide Annotation for Mouse.

[35] Carlson M. (2015). GO.db: A Set of Annotation Maps Describing the Entire Gene Ontology.

[36] Zhu Y., Davis S., Stephens R., Meltzer P.S., Chen Y. (2008). GEOmetadb: Powerful alternative search engine for the Gene Expression Omnibus. Bioinformatics.

[37] Sean D., Meltzer P.S. (2007). GEOquery: A bridge between the Gene Expression Omnibus (GEO) and BioConductor. Bioinformatics.

[38] Szklarczyk D., Morris J.H., Cook H., Kuhn M., Wyder S., Simonovic M., Santos A., Doncheva N.T., Roth A., Bork P. (2017). The STRING database in 2017: Quality-controlled protein–protein association networks, made broadly accessible. Nucleic Acids Res..

[39] Langfelder P., Horvath S. (2008). WGCNA: An R package for weighted correlation network analysis. BMC Bioinform..

[40] Langfelder P., Luo R., Oldham M.C., Horvath S. (2011). Is my network module preserved and reproducible?. PLoS Comput. Biol..

[41] Xu K., Tang C., Tang R., Ali G., Zhu J. A Comparative Study of Six Software Packages for Complex Network Research. Proceedings of the 2010 Second International Conference on Communication Software and Networks.

[42] Ritchie M.E., Phipson B., Wu D., Hu Y., Law C.W., Shi W., Smyth G.K. (2015). limma powers differential expression analyses for RNA-sequencing and microarray studies. Nucleic Acids Res..

[43] Yu G., Wang L.G., Han Y., He Q.Y. (2012). clusterProfiler: An R Package for Comparing Biological Themes among Gene Clusters. OMICS J. Integr.e Biol..

[44] Ahmed M., Kim D.R. (2018). pcr: An R package for quality assessment, analysis and testing of qPCR data. PeerJ.

[45] R Core Team (2017). R: A Language and Environment for Statistical Computing.

[46] Huber W., Carey V.J., Gentleman R., Anders S., Carlson M., Carvalho B.S., Bravo H.C., Davis S., Gatto L., Girke T. (2015). Orchestrating high-throughput genomic analysis with Bioconductor. Nat. Methods.

[47] Merkel D. (2014). Docker: Lightweight Linux containers for consistent development and deployment. Linux J..

